# Bioactive Profile and Rheological Characteristics of Breadfruit (*Artocarpus altilis*) Pulp Commercial Flour

**DOI:** 10.3390/molecules30183732

**Published:** 2025-09-13

**Authors:** Patryk Słota, Ewa Pejcz, Joanna Harasym

**Affiliations:** 1 Adaptive Food Systems Accelerator-Science Centre, Wroclaw University of Economics and Business, Komandorska 118/120, 53-345 Wroclaw, Poland; patryk.slota@ue.wroc.pl (P.S.); joanna.harasym@ue.wroc.pl (J.H.); 2 Department of Biotechnology and Food Analysis, Wroclaw University of Economics and Business, Komandorska 118/120, 53-345 Wroclaw, Poland

**Keywords:** breadfruit flour, *Artocarpus altilis*, gluten-free, alternative flours, techno-functional properties, rheological behavior

## Abstract

The growing demand for sustainable, gluten-free alternatives has positioned breadfruit (*Artocarpus altilis*) flour as a promising multifunctional ingredient. This study comprehensively evaluated commercial breadfruit flour from Mauritius using advanced analytical techniques, including volatiles analysis, Fourier-Transform Infrared Spectroscopy, X-ray diffraction, and rheometry. The flour exhibited interesting techno-functional properties with significant water-binding capacity (6.18 ± 0.45 H_2_O/g DM) and concentration-dependent pasting behavior, achieving 17.895 mPa·s peak viscosity at 16% suspensions with elastic-dominated gel characteristics. Notably, FT-IR spectral analysis revealed a high similarity (95.68%) to acarbose (GLUCOBAY), warranting further biochemical investigation. The flour demonstrated superior oxidative stability (46.3 h) and significant antioxidant capacity, with methanolic extracts yielding 2.27 ± 0.31 mg GAE/g phenolic content and enhanced radical scavenging activities against 2,2-diphenyl-1-picrylhydrazyl (DPPH) 2.81 ± 0.23 μg TE/g and 2,2′-azino-bis(3-ethylbenzothiazoline-6-sulfonic acid) (ABTS) 31.24 ± 0.26 μg TE/g. These findings establish breadfruit flour as a multifunctional ingredient combining excellent technological properties, exceptional oxidative stability, and bioactive applications, positioning it as a valuable sustainable alternative for gluten-free products and functional foods targeting metabolic health management.

## 1. Introduction

Breadfruit (*Artocarpus altilis*), a member of the Moraceae family, is a tropical tree native to the Pacific Islands and widely cultivated in the Caribbean, Southeast Asia, Africa, and Central America [1,2]. Its starchy fruit has long been recognized as a traditional staple food in many tropical regions due to its high carbohydrate content, ease of cultivation, and year-round productivity [3]. Recent years have witnessed growing global interest in breadfruit as a sustainable and nutritious crop with potential applications in modern food systems, particularly for gluten-free products and functional flours [4]. The increasing demand for alternative, nutrient-rich, and gluten-free flour sources has positioned breadfruit as a promising candidate in the field of food innovation.

Breadfruit flour is rich in complex carbohydrates and dietary fiber while being naturally free from gluten, making it suitable for individuals with celiac disease or gluten sensitivity [5]. Moreover, the fruit provides valuable amounts of essential micronutrients such as potassium, calcium, magnesium, and vitamins [6,7]. The compositional profile of breadfruit varies depending on the cultivar, maturity, geographical location, and post-harvest treatment, which necessitates a detailed physicochemical and functional analysis of each specific flour source [1,8].

Studies have shown that breadfruit flour exhibits useful functional properties, including favorable pasting behavior, foaming capacity, water and oil absorption capacities, and emulsion stability [4,9,10]. These characteristics are critical in food formulation, influencing texture, moisture retention, and product shelf-life. Additionally, breadfruit-derived starches demonstrate varied gelatinization and retrogradation properties, making them suitable for both conventional and novel food applications [11,12].

Beyond its functional versatility, breadfruit has attracted attention for its bioactive compounds, particularly polyphenols and antioxidants. Extracts from breadfruit pulp and peel have demonstrated significant antioxidant activity, which could contribute to the nutraceutical and health-promoting potential of breadfruit-based products [5,13,14]. The processing of breadfruit flour, including drying, grinding, and possible pre-treatments, can significantly impact its nutritional profile, microstructure, and technological behavior [15,16].

Given the growing emphasis on sustainable, plant-based food sources, breadfruit stands out due to its high yield per hectare, minimal input requirements, and potential to enhance food security in tropical regions [1,17]. However, despite these advantages, the commercial application of breadfruit flour remains limited, partly due to insufficient characterization of its structural, functional, and technological properties across different production contexts [4].

This study aimed to perform a comprehensive physicochemical, functional, rheological, and structural analysis of commercially available breadfruit flour sourced from Mauritius. The flour was evaluated in terms of moisture and protein content, particle size distribution, color, odor profile, antioxidant activity, functional parameters (e.g., water holding capacity, oil absorption capacity), pasting and rheological behavior, texture, and structural fingerprinting using mid-infrared spectroscopy (FT-IR) and X-ray diffraction (XRD). The results of this study will help assess the potential of breadfruit flour as a functional, gluten-free ingredient in the food industry and provide a reference point for future comparative analyses.

## 2. Results and Discussion

### 2.1. Raw Material Characterization

The commercial breadfruit flour revealed the moisture content—11.45 ± 0.35%, falling within the typical range reported for commercial flours [18]. The protein content was 3.22 ± 0.1% of dry matter, consistent with values described for breadfruit cultivars of comparable origin and maturity [4,10,13].

**Figure 1:**
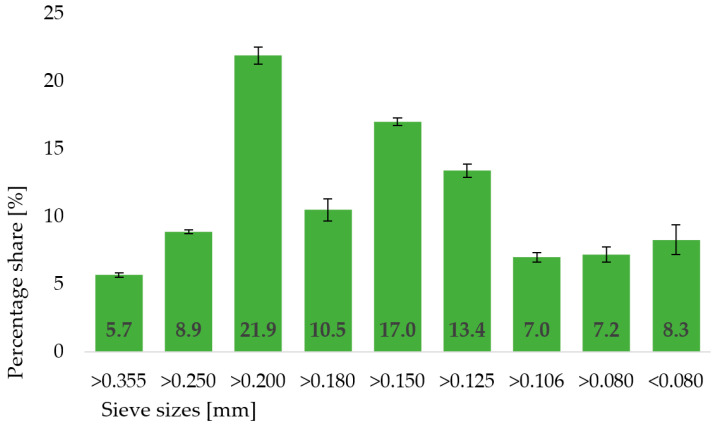
Particle size distribution of commercial breadfruit flour.

Particle size distribution (Figure 1) showed a predominance of fractions between 0.250 and 0.125 mm, reflecting a relatively fine grinding profile and contributing to the good solubility and uniform dispersion of the flour in aqueous systems, important for food processing applications.

The color parameters of breadfruit flour and its water and alcoholic extracts (water, EtOH-Ethanol, MeOH-Methanol) showed distinct differences (Table 1). The flour exhibited the highest lightness (L* = 99.47 ± 2.73), whereas the methanolic extract showed the lowest (95.06 ± 0.10). The flour was strongly yellow and slightly red (a* = 15.37 ± 0.17; b* = 91.48 ± 0.17), while the ethanol and methanol extracts displayed more greenish-blue tones (negative a* values), especially in the methanol extract (a* = −5.02 ± 0.02). The highest chroma (C = 25.19 ± 0.07) was observed in the methanolic extract, indicating greater color saturation. The hue angle (h) of the flour was only 14.91°, confirming warm reddish-yellow tones, whereas the extracts showed much higher h values (>98°). These changes are attributed to solvent-specific extraction of pigmented compounds.

**Figure 2:**
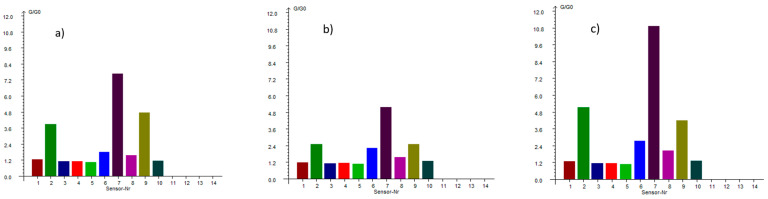
PEN3 sensors response diagram of breadfruit dry flour (**a**) and paste in 2 concentrations: 12% (**b**) and 16% (**c**). Sensor Nr 1: W1C-aromatics, benzene, toluene; 2: W5S-nitrogen oxides, reactive gases; 3: W3C-ammonia, amines; 4: W6S-hydrogen, H_2_S; 5: W5C-propane, methane, aliphatic hydrocarbons; 6: W1S-methane (CH_4_); 7: W1W-H_2_S, sulfur-containing VOCs; 8: W2S-ethanol, CO; 9: W2W-aromatics, organic sulfides; 10: W3S-long-chain alkanes (e.g., hexane, pentane).

Volatile profile analysis showed a domination of aromatic and aliphatic compounds (Figure 2). The change in the volatile profile in pastes demonstrated how thermal treatment and flour concentration alter the aroma of breadfruit products.

The dry flour sample is dominated by the sulfur signature present at G/G0 = 7.891 (sensor 7), confirming breadfruit’s sulfur-rich volatile profile as mainly contributing to its distinctive savory characteristics. However, the behavior of these compounds through processing reveals complex concentration and thermal effects. When the flour undergoes short thermal processing to create the 12% paste, the sulfur compounds initially decrease to G/G0 = 5.168, suggesting typical volatile loss during heating. Yet remarkably, further concentration to 16% paste drives this value to an extraordinary G/G0 = 10.958, the highest reading across all samples and sensors. This non-linear relationship indicates that concentration effects can overcome thermal losses, potentially due to matrix interactions, reduced headspace dilution, or enhanced extraction efficiency in the more concentrated paste system.

The aromatic profile shows a contrasting behavior of progressive decline. The W2W sensor (9), detecting aromatics and organic sulfides, shows a clear downward trend from flour (G/G0 = 4.717) through 12% paste (G/G0 = 2.514) to 16% paste (G/G0 = 1.191). This represents a systematic loss of volatile aromatic complexity, suggesting these compounds are more thermally labile and prone to evaporation during processing.

Nitrogen-containing reactive compounds, measured by the W5S sensor (2) also demonstrate complex profile. Starting at G/G0 = 3.951 in flour, these compounds initially decrease to G/G0 = 2.515 in the 12% paste, consistent with thermal volatilization. Then, in the 16% paste, a recovery of G/G0 = 5.149 is shown, exceeding even the original flour levels. This suggests that higher concentrations may facilitate new formation pathways for reactive nitrogen compounds, possibly through protein degradation, enzymatic activity, or concentration-dependent chemical interactions.

Methane production, detected by the W1S sensor, shows steady increases from flour (G/G0 = 1.858) to 12% paste (G/G0 = 2.224) to 16% paste (G/G0 = 2.751). Similarly, alcohol and carbon monoxide production measured by W2S follows an upward trajectory from flour (G/G0 = 1.492) through 12% paste (G/G0 = 1.575) to 16% paste (G/G0 = 2.033). The thermal processing and concentration create conditions favorable for enzymatic activity, fermentation-like processes, or thermal degradation reactions that generate these metabolic byproducts.

The preservation quality indicators remain consistently low across all samples, with sensors detecting long-chain alkanes, ammonia, and simple aromatics showing minimal responses. From a processing perspective, these results reveal that breadfruit flour processing involves complex trade-offs between different volatile families. The 12% paste represents a middle ground where some thermal losses occur, but the product retains reasonable aromatic complexity while beginning to show concentration benefits. The 16% paste appears to represent an optimal concentration for maximizing sulfur compound intensity, though at the cost of aromatic diversity.

The data suggest that breadfruit’s unique volatile profile is dominated by sulfur chemistry, distinguishing it from many other plant-based flours and pastes. The ability of concentration to enhance certain compound families while diminishing others provides processors with tools to tailor products for specific applications, whether prioritizing complex aromatics or intense savory characteristics.

These results have significant implications for product development, suggesting that different concentration levels could be optimized for different culinary applications, with higher concentrations preferred for applications requiring intense savory notes and lower concentrations maintaining broader aromatic complexity for more delicate applications.

### 2.2. Techno-Functional Properties

The absorptional characteristics of breadfruit flour are shown in Table 2.

The water holding capacity (WHC) of breadfruit flour obtained in the current study was 6.18 ± 0.45 g/g, higher than values reported in previously published studies. For instance, Huang et al. (2019) reported WHC values ranging from 2.04 to 3.32 g/g [19], while Noor et al. (2019) indicated even lower WHC at approximately 1.52 g/g [20]. These considerable differences might result from variations in breadfruit cultivars, geographical origin, maturity stage, growing conditions, and drying methods, all of which significantly influence the physical and chemical characteristics of starch and dietary fiber present in the flour.

The water absorption capacity (WAC) measured in this study was 3.90 ± 0.07 g/g. Unfortunately, the literature data specifically for WAC of breadfruit flour were not available for direct comparison. However, for jackfruit (*Artocarpus heterophyllus*) flour, which is a similar plant, authors indicate WAC values of 2.3 g/g [21]. The relatively high WAC obtained indicates good water absorption potential, likely due to the polysaccharide components and high dietary fiber content in breadfruit pulp.

The obtained oil absorption capacity (OAC) value (2.78 ± 0.08 g/g) was somewhat higher compared to literature data. Huang et al. (2019) reported values between 1.71 and 2.15 g/g [19], whereas Noor et al. (2011) reported a notably lower OAC value of around 0.82 g/g [20]. The discrepancies could be explained by differences in cultivar, particle size, preparation methods of flour, or variations in natural lipid content resulting from the specific parts of fruit used (the flour used in this study was exclusively prepared from pulp without peel or seeds). HLI value over 1 suggests more hydrophilic compounds behavior than lipophilic.

Values obtained for water absorption index (WAI) (9.66 ± 0.14 g/g) and water solubility index (WSI) (9.37 ± 0.85%) represent new data, as literature references for these parameters in breadfruit flour were unavailable. The notably high WAI suggests significant applicability of this raw material, especially in formulations requiring high water retention.

The swelling power (SP) measured was 10.66 ± 0.15 g/g, indicating strong swelling properties of the starch and polysaccharide fractions during heating. A moderate to high swelling capability of breadfruit flour at elevated temperatures was reported [20,22], although exact numerical data are generally not provided. Differences between studies might be due to varying flour preparation methods, specific cultivar properties, fruit maturity stage, and different drying and milling conditions.

The results highlight the beneficial functional properties of the studied breadfruit flour while emphasizing their variability depending on numerous technological, agricultural, and cultivar-specific factors. These variations are particularly relevant given that investigated flour was prepared exclusively from fruit pulp, whereas some literature references might pertain to flours made from whole fruits or other plant parts [23].

### 2.3. Rheological Characteristics of Breadfruit Suspensions and Gels

**Figure 3:**
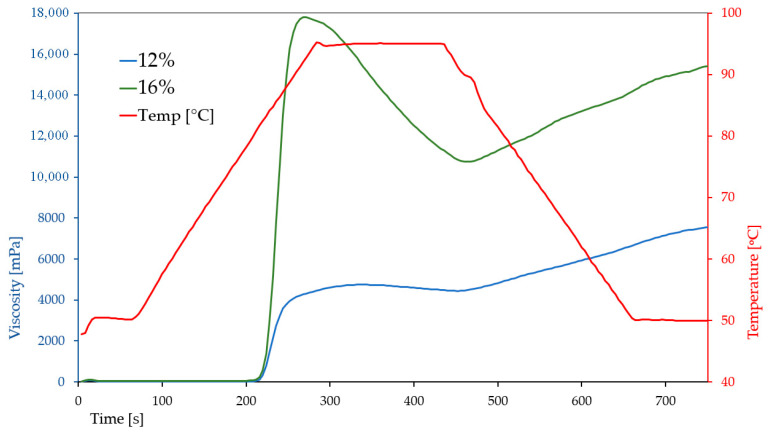
Pasting profiles of breadfruit flour suspensions at 12% and 16%.

The pasting characteristics of breadfruit flour suspensions at two different concentrations (12% and 16%) revealed a strong influence of dry matter content on the rheological properties of the system (Figure 3).

Increasing the flour concentration resulted in markedly higher peak (17.895 ± 260 vs. 4740 ± 126 mPa·s), trough (10.790 ± 94 vs. 4394 ± 106 mPa·s), final (15.533 ± 181 vs. 7642 ± 90 mPa·s), and setback viscosities (4743 ± 114 vs. 3249 ± 43 mPa·s), indicating enhanced starch gelatinization and a stronger tendency for retrogradation (Figure 3). The higher breakdown value at 16% (7105 ± 185 vs. 346 ± 35 mPa·s) suggests greater susceptibility to shear and thermal degradation, most likely due to the increased starch content and swelling capacity at higher concentrations. Peak time was shorter at 16% (4.51 min vs. 5.6 min), indicating faster hydration and granule swelling, whereas the pasting temperature remained stable (~81 °C), reflecting comparable gelatinization thresholds.

These trends are consistent with previous findings by Huang et al. (2020) [22], who reported significantly lower pasting viscosities for breadfruit flour measured at presumably lower concentrations: peak viscosity ranged from 2678 to 2687 mPa·s, final viscosity from 4188.7 to 4310 mPa·s, and setback from 1575 to 1810 mPa·s, with a pasting temperature of ~76.8 °C. The discrepancy in values can be attributed not only to concentration differences but also to cultivar variation, processing methods (e.g., drying technique), and the use of different parts of the fruit. The sample investigated in this study was the flour prepared exclusively from pulp (without peel), which may have altered starch availability and swelling behavior.

Further comparison with the work of Adebowale et al. (2005) [24], who analyzed isolated native breadfruit starch rather than flour, reveals even more pronounced differences. The peak, final, and setback viscosities obtained by those researchers (calculated as 5609, 7701 and 2092 mPa·s, respectively, after conversion from RVU (1 RVU = 12 mPa·s)), were between values obtained in this study for 12% and 16% suspensions. The pasting temperature reported for starch (64.6 °C) was substantially lower than that observed for flour (~81 °C), which is expected due to the absence of fiber, proteins, and other components that typically interfere with starch gelatinization. The use of purified starch in Adebowale’s study, as opposed to whole flour, further highlights the influence of matrix composition on pasting performance and textural potential.

Overall, our results suggest that increasing breadfruit flour concentration considerably enhances its thickening ability and gel strength, making it a promising candidate for use in products such as sauces, puddings, or gluten-free batters where high viscosity and structural integrity are desirable.

**Figure 4:**
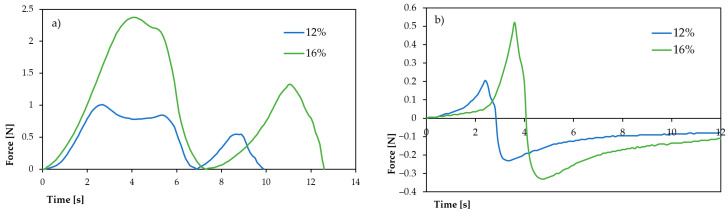
Texture profile (**a**) and stickiness (**b**) of breadfruit flours gels.

The texture profile analysis of gels resulting from cooling the breadfruit flour suspensions of 12% and 16% concentrations revealed substantial concentration-dependent changes in textural characteristics, which strongly correlate with the previously discussed pasting properties (Table 3, Figure 4).

The hardness of breadfruit flour gels increased dramatically from 1.013 ± 0.060 N at 12% concentration to 2.379 ± 0.116 N at 16% concentration, representing a 2.35-fold enhancement. This substantial increase in gel firmness directly correlates with the higher peak viscosity (17.895 vs. 4.740 mPa·s) and storage modulus (G′: 828.49 vs. 639.01 N) observed at higher concentrations. The enhanced hardness reflects the formation of a more robust three-dimensional starch network, consistent with increased starch granule swelling and polymer chain entanglement at higher flour concentrations. This trend aligns with findings by Wang et al. (2011) [12], who reported that breadfruit starch exhibited good gel-forming capacity that improved with concentration.

Cohesiveness values increased significantly from 0.203 ± 0.181 at 12% to 0.346 ± 0.032 at 16%, indicating enhanced internal structural bonding within the gel matrix. The 70% improvement in cohesiveness suggests more extensive hydrogen bonding and physical entanglement between starch chains at higher concentrations. The reduced standard deviation at 16% concentration (±0.032 vs. ±0.181) indicates a more uniform gel structure, likely due to more complete starch gelatinization and consistent network formation throughout the sample.

The springiness parameter demonstrated a moderate but significant increase from 0.754 ± 0.033 to 0.932 ± 0.086, indicating improved elastic recovery of the gel structure after deformation. This 24% enhancement in springiness correlates well with the rheological data showing elastic-dominated behavior (tan δ < 0.3) and suggests that higher concentrations produce gels with better shape retention properties. Such characteristics are particularly valuable for applications requiring structural integrity, such as gluten-free bakery products or shaped food items.

The most crucial textural change was observed in gumminess, which increased four-fold from 0.206 ± 0.031 N to 0.820 ± 0.045 N. Gumminess, calculated as the result of hardness and cohesiveness, reflects the energy required to disintegrate a gel to a state ready for swallowing. This substantial increase indicates that 16% of breadfruit flour gels would require significantly more oral processing, potentially affecting palatability, but also suggesting excellent binding properties for food applications requiring structural stability.

The resilience data shows an apparent inconsistency that warrants discussion. The reported values (2.579 ± 0.171 for 12% vs. 0.951 ± 0.109 for 16%) suggest higher resilience at lower concentration, which contradicts the general trend of improved textural properties at higher concentrations. This may indicate either measurement variability or potential differences in gel maturation between the two concentrations. Further investigation would be needed to clarify this parameter.

Surface stickiness exhibited an inverse relationship with concentration, decreasing approximately fourfold from 0.198 ± 0.018 N at 12% to 0.0519 ± 0.132 N at 16%. This reduction in stickiness is particularly significant for food processing applications, as lower stickiness facilitates handling, molding, and packaging operations. The decreased stickiness at higher concentrations may result from reduced surface moisture availability due to more extensive water binding within the stronger gel matrix, and potentially from different surface tension properties of the more concentrated gel system.

The textural properties strongly correlate with the previously discussed pasting characteristics. The increased hardness and gumminess at 16% concentration align with the higher breakdown viscosity (7.105 vs. 346 mPa·s), suggesting that while the gel is stronger, it may be more susceptible to structural disruption under high shear conditions. The improved cohesiveness and springiness complement the enhanced storage modulus and elastic behavior observed in rheological measurements.

The concentration-dependent textural properties provide valuable insights for food application development. The 12% concentration produces softer, more adhesive gels suitable for applications requiring spreadability or incorporation into soft-textured products. The 16% concentration yields firmer, less sticky gels appropriate for structured food products, meat analogs, or applications requiring shape retention and cutting properties.

While direct literature comparisons for breadfruit flour texture properties are limited, the observed trends are consistent with concentration effects reported for other starch-based systems. The hardness values obtained (1.01–2.38 N) are comparable to those reported for potato and corn starch gels at similar concentrations, suggesting breadfruit flour’s competitive textural performance as an alternative thickening agent.

**Figure 5:**
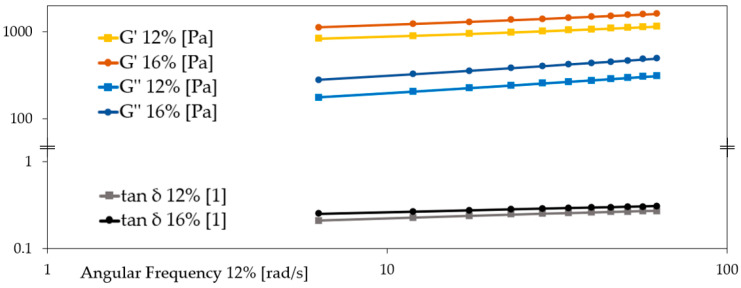
Frequency sweeps rheograms of 12% and 16% breadfruit paste.

The rheological analysis of breadfruit flour pastes at 12% and 16% concentrations revealed a clear influence of dry matter content on the viscoelastic properties of the resulting gels (Table 4; Figure 5). At both concentrations, the storage modulus (G′) was markedly higher than the loss modulus (G″), indicating solid-like, elastic-dominated behavior. This pattern is consistent with the gel-like nature of polysaccharide systems exhibiting a structured three-dimensional matrix.

At 16%, the paste showed a significantly higher G′ (828.49 ± 37.12 N) compared to the 12% sample (639.01 ± 16.77 N), suggesting the formation of a stiffer and more cohesive network. Likewise, G″ increased from 109.63 ± 5.76 N (12%) to 174.72 ± 8.10 N (16%), pointing to enhanced viscous dissipation at higher concentrations. These trends reflect greater starch gelatinization and more extensive entanglement of polymer chains with increasing flour content.

The tan δ (G″/G′) values remained below 0.3 for both samples (0.17 and 0.21), confirming that the elastic component dominated over the viscous one. This aligns with previous findings reported by Wang et al. (2011) [12], who also observed tan δ < 1 in breadfruit starch pastes, attributing it to their strong gel-forming capacity and elastic dominance over viscous flow behavior [12].

Furthermore, the crossover point G′ = G″ occurred at a higher force in the 16% system (561.46 ± 8.33 N) than in the 12% paste (218.24 ± 0.11 N), which confirms the formation of a more robust and deformation-resistant matrix at higher flour content. This parameter, reflecting the structural stability threshold, is often used to define the strength of the gel network.

The observed increase in both G′ and G″ with concentration is also in agreement with the unpublished findings of Huang (2020) [22], who performed oscillatory rheometry on breadfruit flour pastes and noted higher modulus values in denser suspensions. Although no absolute values were reported in that study, the trend supports the current results.

While Wang et al. (2011) [12] focused on isolated starch from breadfruit, and Adebowale et al. (2005) [24] reported RVA and textural data for starch gels, our measurements on whole flour-based systems extend the understanding of breadfruit’s functionality by including the natural matrix of flour (fiber, protein, and residual sugars), which may impact viscoelasticity differently than starch alone.

For instance, the presence of non-starch components may increase energy dissipation (raising G″) or modulate chain interactions (affecting G′), which helps explain the somewhat lower tan δ in your flour-based pastes compared to reported starch gels. Moreover, the fitted model coefficients (a, b, c) from frequency sweep further support consistent elastic behavior across both concentrations, as exponent b remained stable (0.25), suggesting similar frequency-dependence.

### 2.4. X-Ray Diffractograms

**Figure 6:**
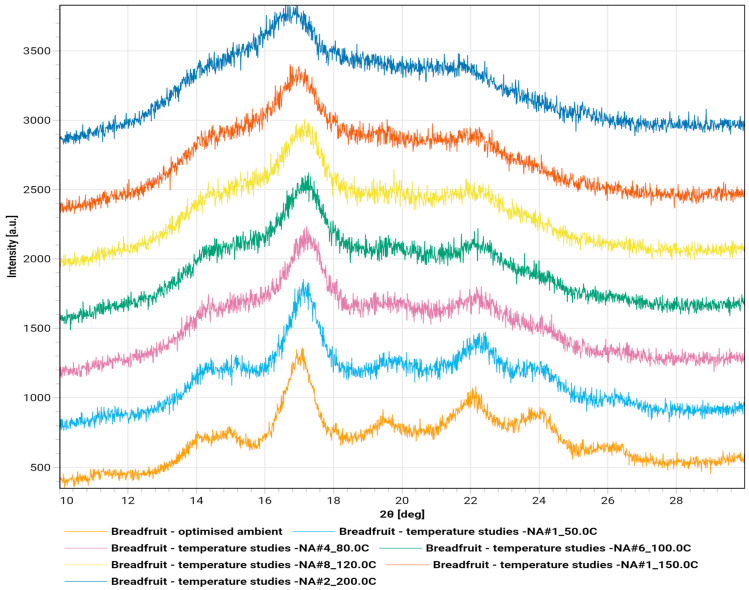
Diffractogram of breadfruit flour in ambient and temperature ramp.

The diffractograms of breadfruit flour in ambient temperature and under thermal stress from 50° C to 200 °C are shown in Figure 6.

The diffractogram revealed a semi-crystalline native starch structure with characteristic A-type polymorph features [9]. The most intense peak at 16.8° 2θ indicates well-ordered crystalline phases with d-spacing of 5.3 Å, typical of cereal and tuber starches [25]. Additional peaks at 18.2° 2θ and 22.5° 2θ confirm the A-type structure, while minor peaks around 20° and 24° 2θ contribute to the crystalline fingerprint [26]. The elevated background and broad underlying hump indicate substantial amorphous content of approximately 63%, typical for native plant flours [27]. The sharp primary peak suggests well-formed crystalline domains with intact starch granules [9]. This A-type polymorph forms a monoclinic crystal structure, distinct from B-type starch found in potato and banana, or C-type starches representing mixed A and B forms [25].

The temperature series demonstrated a systematic transformation from crystalline to amorphous structure during heating [26]. At 50 °C, the diffractogram maintains fundamental peak positions around 15°, 17°, and 23° 2θ with subtle broadening indicating initial long-range order disruption [25]. At 80 °C, peak intensities decreased while widths increased, suggesting significant crystalline domain disruption corresponding to initial starch gelatinization [28]. Hydrogen bonding within crystalline regions weakens due to increased molecular motion and moisture-mediated swelling [27].

100 °C shows substantial crystalline degradation with broader, less intense A-type peaks, indicating extensive loss of long-range order [29]. Elevated background scattering suggests increased amorphous content consistent with advanced gelatinization and crystalline lamellae disruption [27]. At 120 °C, the material exhibits predominantly amorphous character. Sharp crystalline peaks largely disappear, replaced by broad, diffuse scattering patterns representing near-complete loss of original A-type structure with only residual short-range order. 150 °C shows extensive amorphization with very broad, low-intensity scattering [30]. Original crystalline peaks are no longer discernible, indicating complete ordered structure disruption and marking the threshold where crystalline polymorphs convert entirely to amorphous state [31]. 200 °C demonstrates complete structural transformation with only broad, diffuse scattering typical of fully amorphous materials [30]. The absence of crystalline reflections confirms total loss of long-range molecular order, likely accompanied by partial thermal decomposition [31].

The irreversible transformation from native semi-crystalline starch to completely amorphous material shows critical transition occurring between 120 °C and 150 °C [27]. This thermal behavior reflects starch gelatinization followed by complete molecular reorganization, creating an amorphous polymer network unable to revert upon cooling. Progressive peak broadening and intensity loss demonstrate systematic breakdown of hydrogen bonding networks maintaining double-helical arrangements in native A-type starch [27].

### 2.5. Oxidative Stability and Antioxidant Properties

The oxidative stability of the breadfruit flour, determined using the RapidOxy 100 at 80 °C and terminated after a 2% pressure drop, was found to be 46 h and 17 min, indicating high resistance to oxidation. Such a long time is in line with the e-nose response observed earlier, with sensors detecting long-chain alkanes, ammonia, and simple aromatics showing minimal responses. This oxidative stability is significantly higher than values reported for other plant-based products tested with the Rancimat method at higher temperatures (e.g., wheat flour—3.38 h, polenta—2.87 h, dried coconut—3.17 h) [32]. The longer induction time may be due to the mild test conditions, the use of pulp-only flour, as well as the low-fat content of breadfruit. Differences in method (RapidOxy vs. Rancimat) and sample composition must be considered when comparing results. Nonetheless, the outcome confirms breadfruit flour’s suitability for products requiring oxidative stability and extended shelf life.

The antioxidant profile of breadfruit flour revealed significant differences between water and methanolic extraction systems, demonstrating the polarity differentiation of bioactive compounds in breadfruit pulp flour (Table 5).

Total Phenolic Content (TPC) analysis showed that methanolic extraction yielded significantly higher phenolic recovery (2.27 ± 0.31 mg GAE/g DM) compared to aqueous extraction (1.60 ± 0.16 mg GAE/g DM). This 41.9% increase in TPC with methanol extraction aligns with findings reported by Sikarwar et al. (2014) for *A. altilis* leaves, where methanol demonstrated superior phenolic extraction efficiency due to its ability to solubilize diverse polyphenolic structures including flavonoids, phenolic acids, and tannins [5]. The obtained TPC values are comparable to those reported for other tropical fruit flours, such as jackfruit flour (1.89–2.45 mg GAE/g) and banana flour (1.76–2.12 mg GAE/g), confirming breadfruit flour’s position as a phenolic-rich functional ingredient [6,13].

Radical scavenging activities demonstrated consistent patterns across both DPPH and ABTS assays, with methanolic extracts showing markedly superior antioxidant capacity. The DPPH radical scavenging activity increased from 1.72 ± 0.10 μg TE/g DM (water) to 2.81 ± 0.23 μg TE/g DM (methanol), representing a 63.4% enhancement. Similarly, ABTS radical scavenging activity showed even more pronounced differences, with methanolic extracts exhibiting 31.24 ± 0.26 μg TE/g DM compared to 11.37 ± 0.31 μg TE/g DM for aqueous extracts—a remarkable 174.8% increase. This substantial difference in ABTS values suggests that methanol preferentially extracts lipophilic antioxidants that are more effective against the ABTS•+, which can react with both hydrophilic and lipophilic antioxidants, whereas DPPH is limited to lipophilic compounds [30,31].

Ferric Reducing Antioxidant Power (FRAP) showed an interesting inverse relationship, with water extracts demonstrating significantly higher reducing capacity (0.040 ± 0.002 μg FeSO_4_/g DM) compared to methanolic extracts (0.017 ± 0.002 μg FeSO_4_/g DM). This unexpected pattern suggests that water-soluble reducing compounds, possibly including ascorbic acid derivatives, simple phenolic acids, or reducing sugars, contribute substantially to the electron-donating capacity of breadfruit flour. The FRAP assay measures the ability to reduce Fe^3+^ to Fe^2+^, and the higher activity in aqueous extracts indicates the presence of potent water-soluble reductants that may be less efficiently extracted by methanol [32].

Reducing sugar content, determined by the DNS method, was measured only in aqueous extracts (6.58 ± 0.45 μg GluE/g DM), as expected given the hydrophilic nature of sugars. This value reflects the natural sugar content remaining after processing and contributes to both the nutritional profile and potential reducing activity observed in the FRAP assay [29].

These results establish breadfruit flour as a valuable source of natural antioxidants, with the choice of extraction solvent critically determining the profile and potency of recovered bioactive compounds. The synergistic combination of phenolic compounds and water-soluble reductants positions breadfruit flour as a promising ingredient for applications requiring both oxidative stability and health-promoting bioactivity.

### 2.6. Mid-Infrared Spectroscopy

**Figure 7:**
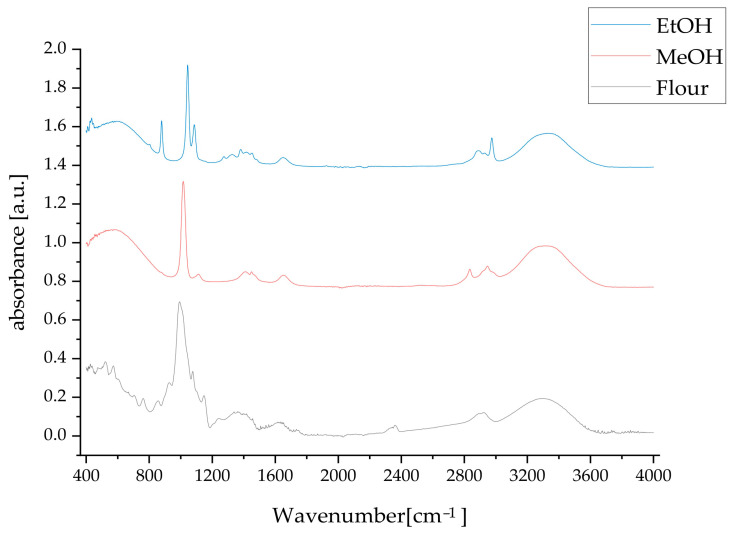
Mid-infrared spectrograms of breadfruit flour and metanolic (MeOH) and ethanolic (EtOH) extracts.

The FT-IR spectral analysis of breadfruit pulp flour and its methanolic and ethanolic extracts reveals distinct compositional differences that reflect the selective extraction capabilities of different solvents and provide insights into the chemical diversity of bioactive compounds present in breadfruit (Figure 7).

The original breadfruit flour displays broad, intense absorption bands at 3067.84, 3301.41, 3317.08 and 3337.28 cm^−1^, characteristic of extensive hydrogen bonding networks in polysaccharides, cellulose, and bound water molecules, which reflects the predominant carbohydrate matrix of the flour, supporting breadfruit flour’s recognition as a healthy, nutrient-dense food option [33].

The methanolic extract shows a more complex O-H stretching pattern with peaks at 3374.18, 3347.65, 3335.70, 3323.76, 3314.47, 3301.21, 3287.94 and 3270.69 cm^−1^. This increased complexity indicates the extraction of diverse phenolic compounds, flavonoids, and hydroxylated secondary metabolites with varying hydrogen bonding environments, suggesting methanol’s ability to extract a broad spectrum of polar bioactive compounds [34].

The ethanolic extract presents the most defined O-H stretching pattern with distinct peaks at 3403.91, 3371.39, 3358.46, 3345.56, 3327.63, 3310.07, 3282.23, and 3278.05 cm^−1^. The sharper, more distinct nature of these absorptions suggests more selective extraction of specific phenolic structures, indicating ethanol’s preference for particular classes of hydroxylated compounds.

The flour spectrum shows minimal absorption in this region, consistent with its predominantly carbohydrate composition. The methanolic extract displays extensive C-H stretching bands throughout the 2400–2600 cm^−1^ range, with notable peaks at 2674.93, 2631.14, 2624.51, and 2616.55 cm^−1^, indicating extraction of complex organic molecules including terpenoids and fatty acid derivatives. In contrast, the ethanolic extract shows a much simpler pattern with primarily one significant peak at 2973.26 cm^−1^, suggesting more selective extraction of compounds with specific alkyl functionalities. This demonstrates ethanol’s preferential extraction of certain lipophilic compounds while being less effective at extracting the full range of aliphatic compounds extracted by methanol. FT-IR spectroscopy combined with chemometric analysis has proven particularly effective for analyzing flavonoids and other bioactive compounds in medicinal plant extracts [35].

The flour spectrum shows minimal carbonyl absorption, reflecting the absence of significant ester or carboxylic acid functionalities in the native carbohydrate matrix. The methanolic extract exhibits prominent carbonyl absorptions at 1785.94, 1771.35, 1762.06 and 1746.14 cm^−1^, indicating extraction of phenolic esters, flavonoid acetates, and other carbonyl-containing secondary metabolites. The ethanolic extract displays carbonyl bands at 1795.86, 1781.72, 1773.11, 1753.45 and 1742.89 cm^−1^, with relatively high wavenumber positions suggesting strained or conjugated carbonyl systems. This indicates ethanol’s ability to extract specific esterified compounds, potentially with different structural characteristics than those extracted by methanol.

A remarkable distinction appears in the ethanolic extract with a significant absorption at 2237.47 cm^−1^, absent in both the flour and methanolic extract. This peak, characteristic of nitrile (C≡N) or acetylenic (C≡C) stretching vibrations, suggests the selective extraction of specialized secondary metabolites such as alkaloids or cyanogenic glycosides, highlighting ethanol’s unique extraction capabilities for certain nitrogen-containing or triple-bond-containing compounds.

The flour spectrum’s fingerprint region (below 1500 cm^−1^) is dominated by polysaccharide-characteristic absorptions, with the strongest peak at 667.99 cm^−1^ representing glycosidic linkages and carbohydrate skeletal vibrations. Both extracts show significantly reduced absorption in the traditional carbohydrate regions (1000–1100 cm^−1^), confirming successful separation of bioactive compounds from the starch matrix.

The methanolic extract displays the most complex fingerprint pattern with numerous overlapping peaks, reflecting the diverse array of extracted compounds. The ethanolic extract shows a more selective pattern with distinct absorptions, particularly in the aromatic regions around 1500–1600 cm^−1^, indicating extraction of specific classes of aromatic compounds. The FT-IR spectral analysis of breadfruit pulp flour and its methanolic and ethanolic extracts reveals distinct compositional differences that reflect the selective extraction capabilities of different solvents, as FT-IR spectroscopy has proven highly effective for characterizing bioactive compounds in herbal medicines [36].

The comprehensive analysis reveals that both solvents successfully extract valuable bioactive compounds from breadfruit flour, but with complementary selectivity patterns that could be strategically utilized depending on the intended application, whether for broad-spectrum nutraceutical use or more targeted therapeutic applications.

**Figure 8:**
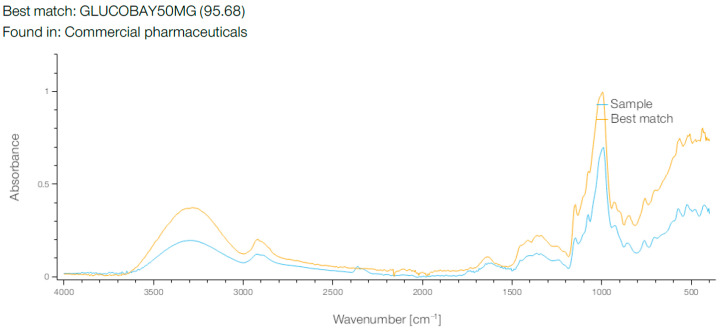
FT-IR Spectrum of Breadfruit flour compared with best match from base—Glucobay 50 mg.

When the breadfruit flour FT-IR spectrum was compared against a pharmaceutical reference database, an unexpectedly high similarity (95.68%) was observed with the spectrum of GLUCOBAY 50 mg (acarbose) (Figure 8). This spectral match represents a novel analytical observation that warrants further investigation. However, several important limitations must be considered when interpreting this finding. Spectral similarity does not indicate identical molecular composition or biological activity, as FT-IR matches reflect similar functional group arrangements rather than specific bioactive compounds. The observed similarity may result from shared carbohydrate structural features rather than active pharmaceutical ingredients, and no direct correlation between spectral similarity and alpha-glucosidase inhibitory activity can be inferred from this spectral data alone.

This FT-IR spectral match between breadfruit flour and GLUCOBAY 50 mg (acarbose) with a remarkable 95.68% similarity is a highly significant finding that provides compelling evidence for the potential antidiabetic properties of breadfruit flour. GLUCOBAY contains acarbose, an alpha-glucosidase inhibitor used clinically to manage type 2 diabetes by delaying carbohydrate digestion and reducing postprandial blood glucose spikes. The exceptionally high spectral match suggests that breadfruit flour contains compounds with very similar molecular structures and functional groups to acarbose, indicating the presence of natural alpha-glucosidase inhibitory compounds.

This finding is particularly noteworthy when considered alongside our previous FT-IR analysis, which revealed the complex carbohydrate matrix of breadfruit flour with characteristic glycosidic linkages at 667.99 cm^−1^ and polysaccharide-related absorptions. The structural similarity to acarbose suggests that some of these carbohydrate-related compounds in breadfruit flour may function as natural enzyme inhibitors, mimicking the pharmaceutical’s mechanism of action.

The spectral overlay shows remarkable convergence across multiple regions, particularly in the fingerprint region below 1500 cm^−1^ and the carbohydrate-characteristic regions around 1000–1200 cm^−1^. This suggests that breadfruit flour naturally contains oligosaccharide or polysaccharide structures that share key functional group arrangements with acarbose’s pseudotetrasaccharide structure.

This discovery has profound implications for breadfruit’s potential as a functional food or nutraceutical, supporting research showing breadfruit extracts can protect pancreatic function in diabetic models [35]. This unexpected spectral relationship suggests that breadfruit flour contains structural motifs similar to those present in acarbose formulations. While intriguing, this finding requires extensive biochemical validation before any functional conclusions can be drawn. Future investigations should include direct alpha-glucosidase inhibition assays, detailed structural elucidation of the compounds responsible for spectral similarity, comprehensive in vitro and in vivo bioactivity studies, and identification of specific molecular structures contributing to the observed spectral match.

The spectral similarity represents a preliminary analytical observation that may guide future research into breadfruit flour’s potential bioactive properties. Nevertheless, extensive validation through appropriate biochemical and biological assays would be required to establish the functional significance of this spectral relationship.

## 3. Materials and Methods

### 3.1. Material

The breadfruit (*A. altilis*) flour used in this study was obtained from Conserverie Sarjua Internationale Ltee (Plaine Lauzun, Port Louis, Mauritius). It is a commercially available product on the local Mauritian market. Due to the lack of detailed information provided by the manufacturer, the specific drying, milling, and raw material processing conditions remain unknown. Upon delivery, the flour was stored in tightly sealed containers at room temperature, in a dry and shaded environment, until further analysis.

### 3.2. Raw Material Characterization

Moisture content was determined using a moisture analyzer ATS60 (AXIS, Gdańsk, Poland) at 105 °C. Approximately 1.0 g of breadfruit flour was placed in aluminum weighing pans and dried to a constant weight. Total protein content was measured using the Kjeldahl method in accordance with AOAC 920.87. Approximately 0.5 g of sample was digested in concentrated sulfuric acid with a catalyst, followed by distillation and titration. A nitrogen-to-protein conversion factor of 5.7 was used.

Color parameters were assessed in the CIELAB system, recording L* (lightness), a* (red/green), and b* (yellow/blue) coordinates. Chroma (C*) and hue angle (*h*°) were calculated as follows:

(1) $$Hue\;\left(h{}^{\circ}\right)=arctan\left(\frac{b^{*}}{a^{*}}\right)$$

(2) $$Chroma\;\left(C^{*}\right)=\sqrt{{a*}^{2}+{b*}^{2}}$$

The color of the flour was measured using a CR-300 colorimeter (Minolta, Tokyo, Japan) in reflectance mode with D65 illumination and a 10° observer angle. The color of the extracts was measured spectrophotometrically using a Vista VTS01166 spectrophotometer (HunterLab, Reston, VA, USA) under the same D65/10° conditions. All measurements were performed in triplicate, and results were expressed as mean ± SD [37].

The volatile profile of breadfruit flour and pastes (12% and 16% dry matter) was evaluated using a PEN 3 electronic nose (AIRSENSE Analytics GmbH, Schwerin, Germany) equipped with ten metal oxide sensors, each with specific sensitivities to different groups of volatile organic compounds (VOCs). The samples showed distinct differences in aroma profiles, which were visualized through radar plots of relative sensor responses (G/G_0_ and G_0_/G). The sensor assignments were as follows:

1. W1C—aromatics (benzene, toluene)
2. W5S—nitrogen oxides and other reactive gases
3. W3C—ammonia and amines
4. W6S—hydrogen and hydrogen sulfide (H_2_S)
5. W5C—propane, methane, aliphatic hydrocarbons
6. W1S—methane (CH_4_)
7. W1W—sulfur-containing compounds (e.g., H_2_S)
8. W2S—ethanol, carbon monoxide
9. W2W—aromatics and organic sulfides
10. W3S—long-chain alkanes (e.g., hexane, pentane).

Breadfruit flour dry sample (1.5 g) or paste (5.0 g) was sealed in glass vials and incubated at 30 °C for 30 min to release volatile compounds. Pasted samples were obtained according to method described in Section 3.4. Sensor response profiles were used to compare aroma characteristics between samples [37].

FT-IR spectra of the samples were recorded using an FT-IR Lyza 7000 spectrophotometer (Anton Paar, Graz, Austria) equipped with an ATR (attenuated total reflectance) accessory with a diamond crystal. The flour and alcoholic extracts (prepared by mixing 0.5 g of flour with 10 mL of 80% methanol/ethanol (99.8% Stanlab, Lublin, Poland) were analyzed. Prior to the measurement, the flour samples were frozen at −70 °C and subsequently lyophilized for 23 h using a freeze dryer Alpha 1-4 LSCplus (Martin Christ Gefriertrocknungsanlagen GmbH, Osterode am Harz, Germany).

Particle size distribution of the breadfruit flour was assessed using an LPzE-2 vibratory sieve shaker (Multiserw Morek, Marcyporęba, Poland), following the AACC Method 66-20.01 with slight modifications [15]. Approximately 50 g of sample was placed on a stack of sieves with mesh sizes ranging from 0.080 to 0.355 mm and shaken for 10 min at a constant vibration amplitude. The proportion of material retained on each sieve was expressed as a percentage of the total sample weight. All measurements were carried out in triplicate.

X-ray diffraction (XRD) analysis was performed to assess the phase composition and thermal structural stability of breadfruit flour samples. Measurements were carried out using a Bruker D8 Advance diffractometer (Bruker AXS, Karlsruhe, Germany), equipped with a Cu Kα radiation source (λ = 1.5406 Å), Göbel mirror, and LynxEye 1D detector. The operating voltage and current were set to 40 kV and 40 mA, respectively. Scans were acquired in the range of 3–80° 2θ with a step size of 0.01° and an acquisition time of 15 min per scan. For the variable temperature analysis, the sample was heated from 30 °C to 200 °C in 10 °C intervals using an Anton Paar HTK1200N heating chamber in a nitrogen atmosphere, with an equilibration time of 5 min at each temperature. A polycrystalline Al_2_O_3_ sample holder was used to minimize background scattering. Quantitative analysis of crystallinity loss was performed using the Bruker DIFFRAC.SUITE EVA v5.2 software, and thermally resolved diffractograms were further evaluated using OriginPro 2024 for graphical interpretation.

### 3.3. Techno-Functional Properties

The functional properties of breadfruit flour, including water holding capacity (WHC), water absorption capacity (WAC), oil absorption capacity (OAC), hydrophilic-lipophilic index (HLI), water absorption index (WAI), water solubility index (WSI), swelling power (SP), foaming capacity (FC), foam stability (FS), emulsifying activity (EA), and emulsion stability (ES), were evaluated according to previously reported procedures [15,38,39].

Water holding capacity (WHC): Approximately 0.5 g (dry basis) of flour was placed in 50 mL falcon tubes with 10 mL of distilled water and left at room temperature for 24 h without agitation. After decanting the supernatant, the hydrated samples were weighed. WHC was calculated as the grams of water retained per gram of dry sample [15].

Water and oil absorption capacity (WAC, OAC)—0.5 g of dry sample was mixed with 10 mL of distilled water or rapeseed oil. The mixtures were vortexed three times for 30 s with 10 min intervals. Samples were then centrifuged at 3000 rpm for 25 min. Excess liquid was decanted and the sediments were weighed. Results were expressed in g of water or oil per g of dry sample. HLI was calculated as the ratio of WAC to OAC, indicating the relative preference for water versus oil binding [40].

Water absorption index (WAI), water solubility index (WSI), and swelling power (SP): Flour suspensions (0.5 g in 10 mL water) were heated in a water bath (MLL147, AJL Electronics, Kraków, Poland) at 90 °C for 10 min and then centrifuged (3000 rpm, 10 min). The supernatants were separated, dried at 130 °C for 3 h (SML, Zalmed, Łomianki, Poland), and weighed. Based on the mass of the sediment and the dried solubles, the indices were calculated using standard formulas [40].

### 3.4. Pasting and Structural Properties of RVA Paste

The pasting characteristics of breadfruit flour was evaluated using a Rapid Visco Analyzer RVA 4500 (Perten Instruments, Stockholm, Sweden) according to the AACC International Method 76-21.01, ICC Standard No. 162 [40]. Two paste concentrations were prepared by combining: 3.4 g of flour with 25.1 g of distilled water for the 12% paste, and 4.58 g of flour with 24.36 g of water for the 16% paste (at a constant moisture content of 14%). The testing cycle included an initial holding phase at 50 °C, followed by controlled heating to 95 °C and subsequent cooling to 50 °C. The total test duration was 13 min. The paddle speed was set at 960 rpm for the first 10 s, and then reduced to 160 rpm for the remainder of the test.

Recorded parameters included peak viscosity, trough, breakdown, final viscosity, setback, and pasting temperature. Additionally, a modified high-solids sample (16% d.m.; 4.58 g flour and 25.1 g water) was analyzed under the same protocol to evaluate the effect of flour concentration on pasting and rheological behavior.

The texture of gels resulting from RVA-derived pastes cooling was assessed using a double compression test (Texture Profile Analysis, TPA) [40] on a texture analyzer FC200STAV200 (AXIS, Kraków, Poland). Cylindrical samples (20 mm height, 20 mm diameter) were compressed twice using a 50 mm diameter flat cylindrical probe. The compression depth was 40% of the initial height at a crosshead speed of 100 mm/min, with a 3 s interval between cycles. Samples were stored at 8 °C for 24 h before measurement. The following parameters were analyzed: hardness, springiness, gumminess, and stickiness. Each measurement was performed in six replicates.

Surface stickiness of the RVA paste was evaluated by a single compression test using the same texture analyzer (FC200STAV200, AXIS, Kraków, Poland) equipped with a 20 mm diameter flat acrylic probe. The samples were analyzed immediately after the RVA run, at an approximate temperature of 40–30 °C following brief cooling.

The probe was lowered to a depth of 5 mm at a speed of 1.0 mm/s, held in contact with the paste for 2 s, and then retracted at the same speed. The maximum force required to detach the probe from the sample, expressed in newtons (N), was recorded as a measure of stickiness. Four replicates were performed.

Rheological characteristics [40] were analyzed using a rotational rheometer MCR 102 (Anton Paar, Graz, Austria) with a parallel plate configuration (25 mm diameter, 1 mm gap) at 25 °C. Sample obtain with 3.4.method was placed on the bottom plate and allowed to stabilize for 5 min. The edges were sealed with paraffin oil to prevent dehydration.

A frequency sweep test (0.1–10 Hz) was conducted at constant strain (0.5%), followed by an amplitude sweep (0.01–100% strain at 1 Hz) and the crossover point (G′ = G″) was also determined. The elastic modulus (G′), viscous modulus (G″), and loss tangent (tan δ) were recorded. Each measurement was conducted in triplicate using RheoCompass software.

### 3.5. Stability and Antioxidant Properties

Oxidative stability of the samples was assessed using a RapidOxy 100 instrument (Anton Paar, Graz, Austria). Precisely 5.00 g of the sample was placed into a sealed test chamber. The analysis was conducted at 80 °C under an initial pressure of 700 kPa using pure oxygen. The endpoint of the test was defined as a 2% drop in pressure relative to the initial value, indicating the onset of oxidative degradation.

To extract antioxidant compounds, 1 g of the sample was mixed with 10 mL of either distilled water or 80% ethanol (Ethanol 99.9%, Stanlab, Lublin, Poland) and agitated for 1 h at room temperature using a laboratory orbital shaker (WU4, Premed, Marki, Poland). The mixture was then centrifuged for 10 min at 3500× *g* (MPW-350, MPW, Warsaw, Poland), and the supernatant was collected for further analysis.

Total phenolic content (TPC) was determined using the Folin–Ciocalteu method [41]. A 20 μL aliquot of the extract was mixed with 100 μL of Folin–Ciocalteu reagent (Chempur, Piekary Śląskie, Poland) and 1.58 mL of distilled water. After incubation at room temperature for 5–8 min, 300 μL of saturated Na_2_CO_3_ solution (Honeywell Specialty Chemicals Seelze GmbH, Seelze, Germany) was added. The mixture was incubated for 30 min at 38 °C (MLL147, AJL Electronics, Kraków, Poland), and absorbance was measured at 765 nm (UV-Vis Ultrospec 2000, Pharmacia Biotech, Piscataway, NJ, USA). TPC was expressed as mg gallic acid equivalent (GAE) (Acros Organics, Bridgewater, NJ, USA) per g dry matter (DM), based on a standard curve.

Reducing sugar content was measured using a modified dinitrosalicylic acid (DNS) method [42]. Briefly, 0.5 mL of the extract was combined with 0.25 mL of DNS reagent (1% 3,5-dinitrosalicylic acid in 0.4 M NaOH, Sigma-Aldrich Chemie GmbH—Steinheim, Germany) and incubated in a boiling water bath for 5 min. After cooling to 50–60 °C, 3 mL of distilled water was added. Absorbance was measured at 530 nm, and results were expressed as μg glucose equivalent (Chempur, Piekary Śląskie, Poland) (GE) per g DM.

Antioxidant activity was evaluated using several assays.

In the DPPH (2,2-diphenyl-1-picrylhydrazyl) assay [43], 34.5 μL of the extract was added to 1000 μL of 0.1 mM DPPH solution in methanol (Sigma-Aldrich Chemie GmbH—Steinheim, Germany) (absorbance 0.9 ± 0.1 at 517 nm). After 20 min of incubation at room temperature, absorbance was read at 517 nm. Results were expressed as μg Trolox equivalent (Sigma-Aldrich Chemie GmbH—Steinheim, Germany) (TE) per g DM.

The ABTS•+ (2,2′-azino-bis(3-ethylbenzothiazoline-6-sulfonic acid)) radical scavenging capacity was assessed based on Sridhar et al.’s protocol [44]. ABTS•+ working solution (Sigma-Aldrich, St. Louis, MO, USA), diluted in phosphate buffer, was mixed with the extract. Absorbance reduction was measured, and results were expressed as μg TE/g DM.

The ferric reducing antioxidant power (FRAP) was determined using a modified method of Re et al. [45]. One milliliter of FRAP working reagent was combined with the extract, and after incubation at 36 °C for 20 min, absorbance was measured at 593 nm (SEMCO, S91 E, Warsaw, Poland). Results were given as μg FeSO_4_·7H_2_O equivalent (Chempur, Piekary Śląskie, Poland) per g DM.

## 4. Conclusions

This study characterizes commercial breadfruit (*A. altilis*) flour and demonstrates the functional and bioactive properties relevant to gluten-free food applications. The flour showed favorable techno-functional characteristics, including substantial water-binding capacity and concentration-dependent rheological behavior with elastic-dominated gel properties suitable for thickening applications.

FT-IR spectral analysis revealed high similarity to acarbose (GLUCOBAY), an observation requiring biochemical validation through alpha-glucosidase inhibition assays. The flour exhibited adequate oxidative stability and moderate antioxidant capacity, with methanolic extracts showing higher phenolic content and radical scavenging activities than aqueous extracts. X-ray diffraction indicated an A-type crystalline structure with thermal transformation occurring between 120 and 150 °C, while volatiles analysis identified a sulfur-dominated profile.

The results indicate that breadfruit flour functions as a multifunctional ingredient with appropriate technological properties, oxidative stability, and bioactive compounds. These characteristics, combined with the sustainability advantages of breadfruit cultivation, suggest potential for commercial applications in products requiring gelling and thickening properties. Further research, including enzymatic assays, sensory evaluation, and food application trials, is needed to establish practical applications in functional food development.

## Figures and Tables

**Table 1:** Chroma analysis of breadfruit flour and its alcoholic extracts.

Parameter	Flour	Extracts		
		Water	EtOH	MeOH
L*	99.47 ± 2.73 ^c^	97.73 ± 0.06 ^b^	99.28 ± 0.04 ^c^	95.06 ± 0.10 ^a^
a*	15.37 ± 0.17 ^d^	−1.21 ± 0.01 ^a^	−2.97 ± 0.02 ^b^	−5.02 ± 0.02 ^c^
b*	91.48 ± 0.17 ^d^	7.86 ± 0.00 ^a^	9.02 ± 0.04 ^b^	24.69 ± 0.07 ^c^
C	14.69 ± 0.46 ^c^	7.95 ± 0.00 ^a^	9.49 ± 0.04 ^b^	25.19 ± 0.07 ^d^
h	14.91 ± 0.26 ^a^	98.72 ± 0.00 ^b^	108.22 ± 0.07 ^d^	101.51 ± 0.08 ^c^

Solvents: EtOH-ethanol, MeOH-Methanol. Superscript letters indicate statistically significant differences between results (*p* ≤ 0.05); a < b < c < d.

**Table 2:** Techno-functional properties of breadfruit flour.

WHC	WAC	OAC	HLI	WAI	WSI	SP
g H_2_O/g DM	g H_2_O/g DM	g Oil/g DM	-	g H_2_O/g DM	g/100g DM	g H_2_O/g DM
6.18 ± 0.45	3.90 ± 0.07	2.78 ± 0.08	1.40 ± 0.04	9.66 ± 0.14	9.37 ± 0.85	10.66 ± 0.15

**Table 3:** Pasting characteristics of breadfruit flour suspensions; texture profile and stickiness of resulting gels.

**Concentration**	**Peak 1**	**Trough 1**		**Breakdown**		**Final Visc**		**Setback**		**Peak Time**	**Pasting Temp**
	**[mPa·s]**	**[mPa·s]**		**[mPa·s]**		**[mPa·s]**		**[mPa·s]**		**[min]**	**[°C]**
12%	4740 ± 126 ^a^	4394 ± 106 ^a^		346 ± 35 ^a^		7642 ± 90 ^a^		3249 ± 43 ^a^		5.6 ± 0.1 ^b^	81.2 ± 0.5 ^b^
16%	17,895 ± 260 ^b^	10,790 ± 94 ^b^		7105 ± 185 ^b^		15,533 ± 181 ^b^		4743 ± 114 ^b^		4.51 ± 0.03 ^a^	80.52 ± 0.49 ^a^
**Concentration**	**Hardness** **[N]**		**Cohesiveness**		**Springiness**		**Gumminess** **[N]**		**Resilience**		**Stickiness** **[N]**
12%	1.013 ± 0.060 ^a^		0.203 ± 0.181 ^a^		0.754 ± 0.033 ^a^		0.206 ± 0.031 ^a^		2.579 ± 0.171 ^b^		0.198 ± 0.018 ^b^
16%	2.379 ± 0.116 ^b^		0.346 ± 0.032 ^b^		0.932 ± 0.086 ^b^		0.820 ± 0.045 ^b^		0.951 ± 0.109 ^a^		0.0519 ± 0.132 ^a^

12%, 16%—breadfruit flour concentration in suspensions and gels. Superscript letters indicate statistically significant differences between results (*p* ≤ 0.05); a < b.

**Table 4:** Frequency sweep data of breadfruit flour paste in 12% and 16% concentration.

Concentration	G′ [Pa]	a	G″ [Pa]	b	tan δ	c	G′ = G″ [Pa]
12%	639.01 ± 16.77 ^a^	0.14 ± 0.00 ^a^	109.63 ± 5.76 ^a^	0.25 ± 0.00 ^a^	0.17 ± 0.00 ^a^	0.11 ± 0.00 ^b^	218.24 ± 0.11 ^a^
16%	828.49 ± 37.12 ^b^	0.16 ± 0.00 ^b^	174.72 ± 8.10 ^b^	0.25 ± 0.01 ^a^	0.21 ± 0.00 ^b^	0.09 ± 0.01 ^a^	561.46 ± 8.33 ^b^

Superscript letters indicate statistically significant differences between results (*p* ≤ 0.05); a < b.

**Table 5:** Antioxidant and reducing activity, total polyphenol and reducing sugars content.

Solvent	TPC	DPPH	ABTS	FRAP	DNS
	GAE/g DM	TAE/g DM	TAE/g DM	FeSO_4_/g DM	GluE/g DM
Water	1.60 ± 0.16 ^a^	1.72 ± 0.10 ^a^	11.37 ± 0.31 ^a^	0.040 ± 0.002 ^b^	6.58 ± 0.45
Methanol	2.27 ± 0.31 ^b^	2.81 ± 0.23 ^b^	31.24 ± 0.26 ^b^	0.017 ± 0.002 ^a^	-------

GAE-Gallic Acid Equivalent, TAE-Tannic Acid Equivalent, GluE-Glucose Equivalent, Superscript letters indicate statistically significant differences between results (*p* ≤ 0.05); a < b.

## Data Availability

The original contributions presented in this study are included in the article. Further inquiries can be directed to the corresponding author.
